# Selective Binding of Heparin/Heparan Sulfate Oligosaccharides to Factor H and Factor H-Related Proteins: Therapeutic Potential for C3 Glomerulopathies

**DOI:** 10.3389/fimmu.2021.676662

**Published:** 2021-08-18

**Authors:** Markus A. Loeven, Marissa L. Maciej-Hulme, Cansu Yanginlar, Melanie C. Hubers, Edwin Kellenbach, Mark de Graaf, Toin H. van Kuppevelt, Jack Wetzels, Ton J. Rabelink, Richard J. H. Smith, Johan van der Vlag

**Affiliations:** ^1^Department of Nephrology, Radboud Institute for Molecular Life Sciences, Radboud University Medical Center, Nijmegen, Netherlands; ^2^Biochemical Technical Support Aspen Oss, Oss, Netherlands; ^3^Department of Biochemistry, Radboud Institute for Molecular Life Sciences, Radboud University Medical Center, Nijmegen, Netherlands; ^4^Department of Nephrology and Einthoven Laboratory for Vascular Medicine, Leiden University Medical Center, Leiden, Netherlands; ^5^Departments of Internal Medicine and Otolaryngology, Carver College of Medicine, Iowa City, IA, United States

**Keywords:** complement, factor H (FH), factor H-related protein, heparan sulfate (HS), heparin, complement 3 glomerulopathy

## Abstract

Complement dysregulation is characteristic of the renal diseases atypical hemolytic uremic syndrome (aHUS) and complement component 3 glomerulopathy (C3G). Complement regulatory protein Factor H (FH) inhibits complement activity, whereas FH-related proteins (FHRs) lack a complement regulatory domain. FH and FHRs compete for binding to host cell glycans, in particular heparan sulfates (HS). HS is a glycosaminoglycan with an immense structural variability, where distinct sulfation patterns mediate specific binding of proteins. Mutations in FH, FHRs, or an altered glomerular HS structure may disturb the FH : FHRs balance on glomerular endothelial cells, thereby leading to complement activation and the subsequent development of aHUS/C3G. In this study, we aimed to identify specific HS structures that could specifically compete off FHRs from HS glycocalyx (HS_Glx_), without interfering with FH binding. FH/FHR binding to human conditionally immortalized glomerular endothelial cells (ciGEnCs) and HS_Glx_ purified from ciGEnC glycocalyx was assessed. HS modifications important for FH/FHR binding to HS_Glx_ were analyzed using selectively desulfated heparins in competition with purified HS_Glx_. We further assessed effects of heparinoids on FHR1- and FHR5-mediated C3b deposition on ciGEnCs. In the presence of C3b, binding of FH, FHR1 and FHR5 to ciGEnCs was significantly increased, whereas binding of FHR2 was minimal. FHR1 and 5 competitively inhibited FH binding to HS_Glx_, leading to alternative pathway dysregulation. FHR1 and FHR5 binding was primarily mediated by N-sulfation while FH binding depended on N-, 2-O- and 6-O-sulfation. Addition of 2-O-desulfated heparin significantly reduced FHR1- and FHR5-mediated C3b deposition on ciGEnCs. We identify 2-O-desulfated heparin derivatives as potential therapeutics for C3G and other diseases with dysregulated complement.

## Introduction

The complement system serves an important role in immunity by removing pathogens and apoptotic debris. Three distinct pathways can induce complement activation, with the alternative pathway (AP) targeting both pathogens and host tissues. Complement activation results in the cleavage of complement component C3 to C3b, which attaches covalently to cell surfaces and extracellular matrix. C3b recruits complement factor B to form a catalytic complex called C3 convertase that results in feed-forward amplification of complement activation, culminating in the formation of the lytic pore C5b-9 complex (1).

Failure to control AP activation on host tissues can lead to disease, including the glomerular diseases complement component 3 glomerulopathy (C3G) (2) and atypical hemolytic uremic syndrome (aHUS) (3). C3G and aHUS have been associated with genetic variations in various complement genes, including complement factor H (*CFH*), which encodes Factor H (FH) the main inhibitor of the AP (4). FH, which consists of 20 homologous complement control protein (CCP) domains, binds to C3b (5) to displace factor B (decay accelerating activity, DAA) (6) and it also acts as cofactor for complement factor I-mediated cleavage of C3b into iC3b (cofactor activity) (7). FH differentiates between host tissues and pathogens by binding to heparan sulfate (HS) on host tissues using CCP7 (8, 9) and CCP20 (10), and to sialic acid using CCP20 (11). FH-mediated complement control is further modulated by six FH-related proteins (FHR1-3, 4A, 4B and 5) which contain CCPs with high homology to many but not all CCPs of FH. Notably the N-terminal complement regulatory domain present in FH is absent in FHRs. CCPs in FHR proteins that are homologous to CCP7 and CCP19-20 in FH facilitate FHR binding to FH ligands, including HS and C3b (12–14). FHRs have therefore been proposed to compete with FH for ligands on host tissues such as glomerular endothelial cells and the glomerular basement membrane, potentially leading to local dysregulation of AP activation and tissue damage (15).

FHRs can be divided into type 1 FHRs (FHR1, FHR2, and FHR5), which occur as homo- and heterodimers, and type 2 FHRs (FHR3, FHR4A, and FHR4B), which are monomeric in plasma (16, 17). Type 1 FHRs, particularly FHR1 and FHR5, are prominently found in renal biopsies of C3G patients (18). In addition, in both aHUS and C3G patients, pathologic type 1 FHR gene rearrangements have been described that generate novel FHRs proteins with increased abilities to compete with FH for cell surface and extracellular matrix binding (15, 19, 20).

We hypothesized that pathogenic changes, for example genetically inherited, or induced by infections or high blood pressure, in glomerular tissues, *e.g.* deviations in the HS structures, may also affect the balance between FH and FHR binding, thereby further contributing to disease development.

HS is a linear polysaccharide from the glycosaminoglycan family that is particularly prominent in the endothelial glycocalyx, a thick glycan layer lining the lumen of blood vessels (21). HS is synthesized by a complex biomachinery that involves more than 30 enzymes (22). Initially, a carbohydrate backbone is generated by the action of exostosin 1/exostosin 2 (EXT1/2) copolymerase that adds up to 100-200 repeating units of the glucuronic acid-N-acetylglucosamine (GlcA-GlcNAc) disaccharide motif. Subsequently, N-deacetylase/N-sulfotransferases (NDSTs) substitute acetyl groups in GlcNAc residues for sulfates, the first of several different sulfate modifications. GlcA can be converted to iduronic acid (IdoA) by glucuronic acid epimerase (GLCE), introducing additional structural variability (23). Finally, sulfate modifications are added by sulfotransferases (HS2STs, HS3STs, and HS6STs) at the 2-O-position of GlcA/IdoA and the 3-O- and/or 6-O-positions, respectively, of GlcNAc/GlcNS. The number of structural possibilities within an HS chain is immense; considering 48 possible disaccharides, regarding possible combinations of modifications, an average HS chain of 100 disaccharides has 48^100^ which equals 10^168^ possible structures. The structural diversity of HS, mainly dictated by distinct sulfation patterns, explains why HS mediates the specific binding and function of a myriad of proteins, including complement proteins (24, 25). Notably, heparin and highly sulfated domains within HS are similar, whereas heparin overall is more sulfated than HS.

While the cell surface recognition domains of FH and FHRs are highly homologous, they are not identical. It is known that small changes in the HS binding domains of FH significantly affect the specificity of protein interaction with certain sulfate modifications (26), and therefore it is reasonable to hypothesize that similar differences in specificity may exist between FH and FHRs. Theoretically, the relative binding affinities of FH and FHR to host tissues, *i.e.* endothelial glycocalyx, may be different, which could be selectively inhibited by HS or heparinoid oligosaccharides. Therefore, in this study, we sought to investigate whether HS structures can be identified, which differentially alter FH and/or FHR binding to the glycocalyx of glomerular endothelial cells. Indeed, we found that binding of FH, FHR1 and FHR5 proteins to glomerular endothelial HS is differentially mediated by N-, 2-O- and 6-O-sulfation. These findings offer novel insights into the underlying pathophysiology of C3G and may lead to novel treatments for C3G and other diseases with dysregulated complement activity.

## Materials and Methods

### Participants

Patients with C3G referred to the Molecular Otolaryngology and Renal Research Laboratories (MORL) at the University of Iowa (UI) for a genetic evaluation of complement genes were enrolled in this study. C3G was diagnosed by the presence of C3 deposits by immunofluorescence in the absence, or comparatively reduced presence of other immunoreactants (C3 immunofluorescence at least two orders of magnitude greater intensity than for any other immunoreactant). Copy number variation in the *CFH*-*CFHR* genomic region was determined using multiplex ligation-dependent probe amplification. The study was approved by the Institutional Review Board of Carver College of Medicine at the University of Iowa.

### Cell Culture

Conditionally immortalized human glomerular endothelial cells (ciGEnCs) were cultured as previously described (27) on 1.0 µg/cm^2^ bovine fibronectin (Bio-Connect BV, Huissen, The Netherlands) coating. For proliferation, cells were cultured at 33°C in endothelial cell basal medium (EGM-2 MV; Lonza, Verviers, Belgium). For experiments, ciGEnCs were seeded at 25% confluence at 37°C and grown to confluent monolayers. Human umbilical vein endothelial cells (HUVECs) were cultured as previously described (28). Media were refreshed every two days.

### Evaluation of FH/FHR ciGEnC Binding Using ELISA

ciGEnCs were differentiated in 96 well plates for five days. After differentiation, cells were washed twice using Hank’s balanced salt solution including Mg^2+^/Ca^2+^ (HBSS) and incubated for 30 minutes at RT with two-fold dilution series of serum-purified FH (Complement Technologies Inc., Tyler, TX, USA) or recombinant 6xHis-tagged FHR1, FHR2 and FHR5 (Novoprotein, Summit, NJ, USA) in HBSS with 2% (w/v) bovine serum albumin (BSA; Sigma-Aldrich Chemie, Zwijndrecht, The Netherlands). Binding was detected using monoclonal anti-FH antibody (Clone: Ox24; Bio-Rad) followed by goat anti-mouse IgG_1_:horseradish peroxidase (HRP) conjugate (Sanbio BV, Uden, The Netherlands), or mouse anti-6xHis antibody (Sigma-Aldrich) followed by goat anti-mouse IgG : HRP conjugate (Jackson ImmunoResearch, West Grove, PA). Assays were developed using 3, 3’, 5, 5’ tetramethyl benzidine substrate A+B (Biolegend, London, UK). Based on the titrations, FH, FHR1, FHR2 and FHR5 were used at a concentration of 100, 70, 50 and 10 µg/ml for all subsequent assays, unless stated otherwise.

For evaluation of FH and FHR binding to C3b-labeled ciGEnCs, cells were cultured in 96 well plates for five days at 37°C, washed twice with HBSS and sensitized with anti-Jurkat/Ramos/THP-1 antiserum (1:30 in HBSS) (29). Classical pathway activation was then induced by incubation with 3.60 µg/ml C1, 1.00 µg/ml C2, 6.00 µg/ml C4 and 130 µg/ml C3 (Complement technologies Inc.) in HBSS for 20 minutes at 37°C. C3b labeling was confirmed using polyclonal sheep anti-C3 antibody (ICL Inc., Portland, OR, USA) followed by rabbit anti-sheep IgG: HRP conjugate (Sanbio BV). For competition assays between FH and FHRs on C3b-labeled ciGEnCs, ciGEnCs were preincubated with FHRs for 10 minutes before addition of FH for 20 minutes at RT. Binding of FH and FHRs to C3b-labeled ciGEnCs was determined as described above.

### Alternative/Terminal Pathway Activation Assays

ciGEnCs and HUVECs were cultured in 24 or 48 well plates as described above. After differentiation, cells were washed twice with 0.2% (w/v) BSA in phosphate-buffered saline (PBS) and incubated for 30 minutes at 37°C with 20% (v/v) normal human serum (NHS; Complement Technologies Inc., Tyler, TX, USA) in veronal-buffered saline [15 mM barbitone, 145 mM NaCl, 5 mM MgCl_2_, 5 mM EGTA, 0.025% NaN_3_ (pH 7.3)] or 20% NHS supplemented with 35 µg/ml FHR1, 25 µg/ml FHR2 or 5 µg/ml FHR5 in veronal-buffered saline. 20% NHS supplemented with 10 mM EDTA was used to correct for cell surface binding of C3 in absence of complement activation. Monoclonal antibodies specific for decay-accelerating factor (Bio-Rad Laboratories BV, clone: BRIC-216) or membrane cofactor protein (Santa Cruz Biotechnology, Santa Cruz, CA, USA, clone: M177) were included to sensitize ciGEnCs to AP activation. To determine AP dysregulation on ciGEnCs in patient serum, cells were incubated with C3G patient sera including: patients with 3 to 4 copies of the *CFHR1* gene, the *CFHR1* and *CFHR3* gene deletion (Δ*CFHR3*-*CFHR1*), 402H/H or 402Y/Y haplotype. Since C3G patient sera samples were depleted of C3, the sera were supplemented with either 20% NHS or C3 (130 µg/ml), factor B (21 µg/ml) and factor D (0.2 µg/ml) (Complement Technologies Inc.) before addition to the cells. Patient sera samples that did not exceed the C3 signal of NHS supplemented with EDTA (MFI: 54819 ± 6202) by at least 2.5-fold after AP supplementation were rejected from analysis. To determine potential therapeutic effects, FHR-supplemented sera were spiked with 2-O-desulfated heparin (oligosaccharides) before addition to the cells. Cells were detached by incubation with 1% (w/v) BSA in non-enzymatic cell dissociation solution (Sigma-Aldrich Chemie) and gentle scraping, centrifuged at 500g and resuspended in 0.5% (w/v) BSA in ice-cold PBS. C3 and C9 deposition was detected using polyclonal sheep anti-C3 antibodies and goat anti-C9 antiserum (Quidel, San Diego, CA, USA). Antibody binding was quantified with Alexa 488-labeled donkey anti-sheep IgG and donkey anti-goat IgG (Life Technologies, Breda, The Netherlands) using a Beckman Coulter CytoFLEX flow cytometer with Kaluza 2.1 software. To determine if the classical pathway contributes to the observed C3b deposition, cells were sensitized with anti-Jurkat/Ramos/THP-1 antiserum and incubated with 20% NHS in either veronal-buffered saline or HBSS (**Supplementary Figure 1**). To validate the flow cytometry assay used to analyze C3b deposition, C3b deposition was also analyzed using SDS PAA gel electrophoresis and Western blotting.

### Heparan Sulfate Purification Using Low Melting Agarose Barium Acetate Agarose Gel Electrophoresis

Glycosaminoglycan extractions were performed as described previously (30). GAGs were loaded on 1% (w/v) low-melting agarose gel (Boehringer Mannheim, Mannheim, Germany), and resolved by gel electrophoresis in 50 mM barium acetate buffer, pH 5.0 (Sigma-Aldrich). Gels were stained using Azure A (Sigma-Aldrich) and ciGEnC glycocalyx-derived HS (HS_Glx_) bands were collected and melted at 65°C for 10 minutes. After extraction using an equal volume basic phenol (Boom BV, Meppel, The Netherlands), HS_Glx_ in the aqueous phase was collected and HS_Glx_ was precipitated by addition of 0.24 µl 27% (w/v) sodium acetate (Sigma-Aldrich) and 3.72 µl ethanol (Merck, Darmstadt, Germany) per µl aqueous phase, followed by overnight incubation at -20°C. Precipitates were collected by centrifugation at 15,700g for 10 minutes and resuspended in H_2_O. Three consecutive precipitations were performed per HS_Glx_ extract.

### 2-O-Desulfated Heparin Oligosaccharide Library Generation

2-O-desulfated heparin was generated from 100 mg heparin (Aspen Oss BV, Oss, The Netherlands) as described previously (31). A 5% (w/v) solution of heparin in H_2_O was alkalized by adding sodium hydroxide (Merck) to 0.5 M concentration, snap-freezing and lyophilization. 1% (w/v) sodium borohydride (Sigma Aldrich) was included to minimize degradation by β-elimination. The resulting product was dissolved and neutralized by adding 5% (v/v) acetic acid and dialyzed extensively against H_2_O. Selective 2-O-desulfation was confirmed by disaccharide analysis of 4 mg heparin or 2-O-desulfated heparin as described previously (32). 2-O-desulfated heparin oligosaccharide libraries were generated following the protocol described by Powell et al. (33). 40 mg 2-O-desulfated heparin were digested overnight at 37°C using 1.0 mU/mg heparinase II (Iduron, Macclesfield, UK) in 2.0 ml 100 mM acetate (pH 7.0), 1.5 mM calcium chloride. 5.00 to 8.75 mg/separation of 2-O-desulfated heparin digest were resolved by size exclusion chromatography in 0.25 M ammonium bicarbonate at 0.22 mL/min flow speed over a BioGel P10 column (75 x 1.6 cm; Bio-Rad Laboratories BV) while monitoring the absorbance at 232 nm and collecting 1.0 mL fractions. Fractions corresponding to defined peaks were pooled, dialyzed against H_2_O using 100-500 Da molecular weight cut-off Float-A-Lyzers (Repligen, Breda, Netherlands) and then concentrated by centrifugal evaporation. 2-O-desulfated heparin oligosaccharides were quantified using a Smart-Spec Plus spectrophotometer (Bio-Rad Laboratories BV) to measure the absorbance 232 nm and an extinction coefficient of 5500 M^-1^cm^-1^ (34). Successful digestion and size separation were confirmed using polyacrylamide gel electrophoresis compared to Arixtra, (GlaxoSmithKline, Brentford, UK) and size-defined heparin oligosaccharide standards (gifted from Prof. Jerry Turnbull’s lab) (33) and silver-staining following Morrissey (35).

### Competition Assays Using Selectively Desulfated Heparin (Oligosaccharides)

HS_Glx_ was immobilized on microtiter plates (NUNC, Roskilde, Denmark) at a concentration which resulted in ~70% of the EW4G2 (anti-HS antibody) signal obtained from 1.0 µg/well of heparan sulfate from bovine kidney (Sigma-Aldrich). Plates were washed using PBS/0.05% Tween-20 and blocked using 2% BSA in HBSS for 2 hours at RT. Binding of FH, FHR1, FHR2 and FHR5 after 1 hour incubation at RT was determined as described for cell surface binding assays. For competition between FH and FHRs, plates were preincubated with FHR1 or FHR5 in 2% BSA in HBSS for 1 hour at RT before incubation with FH. The role of HS modifications was evaluated by preincubating FH, FHR1 and FHR5 with 100 µg/ml heparin, N-, 2-O- and 6-O-desulfated heparin (Iduron, UK) for 1 hour at RT before addition to immobilized HS_Glx_. To determine the minimum oligosaccharide size required for inhibition of FHR1 and FHR5 binding to HS_Glx_, proteins were preincubated with 10 µM of the different size exclusion chromatography fractions.

### Statistics

Groups were compared with Student’s unpaired t-test or One-way ANOVA (>2 groups) followed by Tukey’s *post-hoc* test using GraphPad Prism 9.1.2 (GraphPad Software Inc., San Diego, CA). Values are given as mean ± standard error of the mean unless stated otherwise. Statistical significance was accepted for *p*≤ 0.05.

## Results

### FH and Type 1 FHRs Bind to (C3b-Labeled) Glomerular Endothelial Cells *In Vitro*

**Figure 1:**
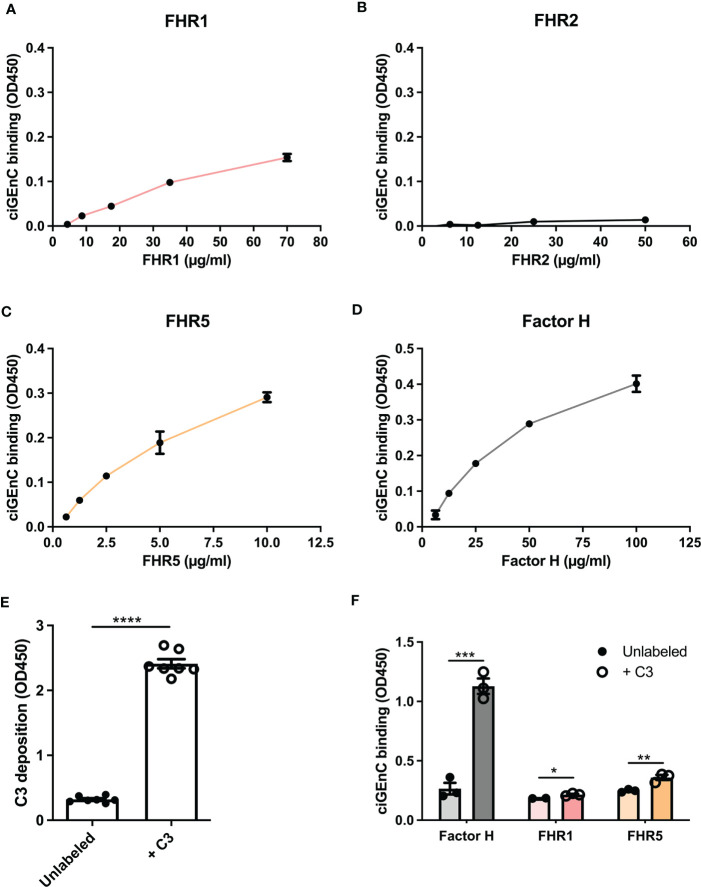
Factor H and recombinant FHR1 and FHR5 bind to glomerular endothelial cells *in vitro.* Factor H-related proteins (FHRs) 1 **(A)**, 2 **(B)** and 5 **(C)** and factor H **(D)** were titrated on conditionally immortalized glomerular endothelial cells (ciGEnCs) and binding was determined using ELISA. Labeling antibody-sensitized ciGEnCs with C3b using classical pathway proteins (n=7) **(E)** significantly increased binding of factor H and FHR1 and FHR5 (n=3) **(F)**. (*p < 0.05, **p < 0.01, ***p < 0.001, ****p < 0.0001 *vs* Unlabeled).

To investigate the role of specific HS/heparinoid sulfate modifications in the interaction between FH, type 1 FHRs and HS in the glomerular endothelial glycocalyx *in vitro*, we first evaluated the binding characteristics of FH and FHRs to ciGEnCs. All proteins, except FHR2, bound to the cell surface within their reported physiological concentrations (13, 36, 37) (**Figures 1A–D**). Notably, since FHR2 hardly binds to ciGEnCs, we mainly focused on FHR1 and FHR5 in our subsequent experiments. Next, we wondered whether the presence of C3b would influence FH and FHRs binding. To this end, we deposited C3b on antibody-sensitized ciGEnCs using classical pathway proteins (**Supplementary Figure 1** and **Figure 1E**), which significantly increased binding of FH and FHRs, though FHR2 binding remained relatively weak compared to FHR1 and FHR5 (**Figure 1F** and **Supplementary Figure 2A**).

### FHR1 and FHR5 Cause Complement Activation on Glomerular Endothelium *In Vitro*

**Figure 2:**
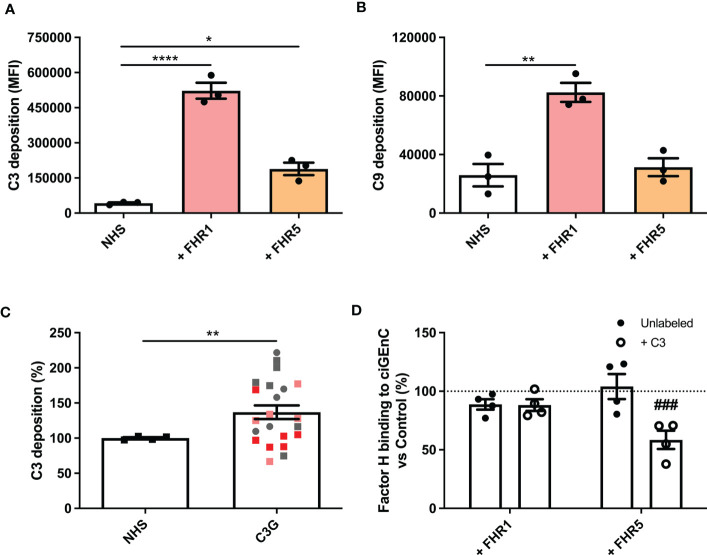
Factor H-related proteins 1 and 5 deregulate alternative pathway activation on glomerular endothelial cells *in vitro*. ciGEnCs were incubated in 20% normal human serum (NHS) in veronal-buffered saline including 5 mM magnesium-EGTA, which prevents classical/lectin pathway activation. Serum was supplemented with factor H-related proteins (FHRs) 1 or 5 and effects on alternative pathway (n=3) **(A)** and terminal pathway (n=3) **(B)** activity were evaluated using flow cytometry. ciGEnCs were incubated with NHS or C3G patient sera (n=4, healthy controls; n=22, C3G patients) **(C)**, including patients: with 3 to 4 copies of the FHR1 gene (pink), FHR1 and FHR3 deletion (ΔCFHR3-1) (red), 402H/H haplotype (grey square) and 402Y/Y haplotype (grey circle). Sera were supplemented with C3, factor B and factor D to ensure the presence of sufficient amounts of AP components. To determine the effect of FHR competition on FH binding, unlabeled or C3b-labeled ciGEnCs were pre-incubated with buffer (Control) or FHRs 1 or 5, after which FH binding was detected using ELISA (n=4) **(D)**. (*p < 0.05, **p < 0.01, ****p < 0.0001 *vs* NHS; ^###^p < 0.001 *vs* Control).

Next, we evaluated the effect of FHRs on complement regulation on ciGEnCs. Supplementing human serum with FHR1, FHR2 or FHR5 before incubation with ciGEnCs revealed significant dysregulation of AP activity, as measured by increased C3b deposition, in the case of FHR1 and FHR5 (**Figure 2A** and **Supplementary Figure 2B**), and the terminal pathway activity, as measured by C9 deposition, in the case of FHR1 (**Figure 2B** and **Supplementary Figure 2C**). Furthermore, when ciGEnCs were incubated with serum from C3G patients, significant dysregulation of AP activity was observed (p=0.0011) compared to healthy control serum (**Figure 2C**). Notably, to validate our flow cytometry assay to measure C3b deposition, we also analyzed C3b deposition in resolved cellular extracts followed by Western blotting, which essentially yielded quantitatively similar results (**Supplementary Figure 3**). To determine if the observed dysregulation of the AP by the added FHRs results from decreased FH binding to ciGEnCs, FH was added to cells after incubation with FHRs. While no significant inhibition of FH binding was observed on untreated ciGEnCs, FHR5 significantly reduced cell surface binding of FH after ciGEnCs were labeled with C3b using the classical pathway proteins (**Figure 2D** and **Supplementary Figure 2D**). Thus, HS-binding of type 1 FHRs deregulates complement activity on ciGEnCs, which may depend in part on C3b deposition.

### FHR1 and FHR5 Compete With FH for Binding to HS Purified From Glomerular Endothelial Glycocalyx (HS_Glx_)

**Figure 3:**
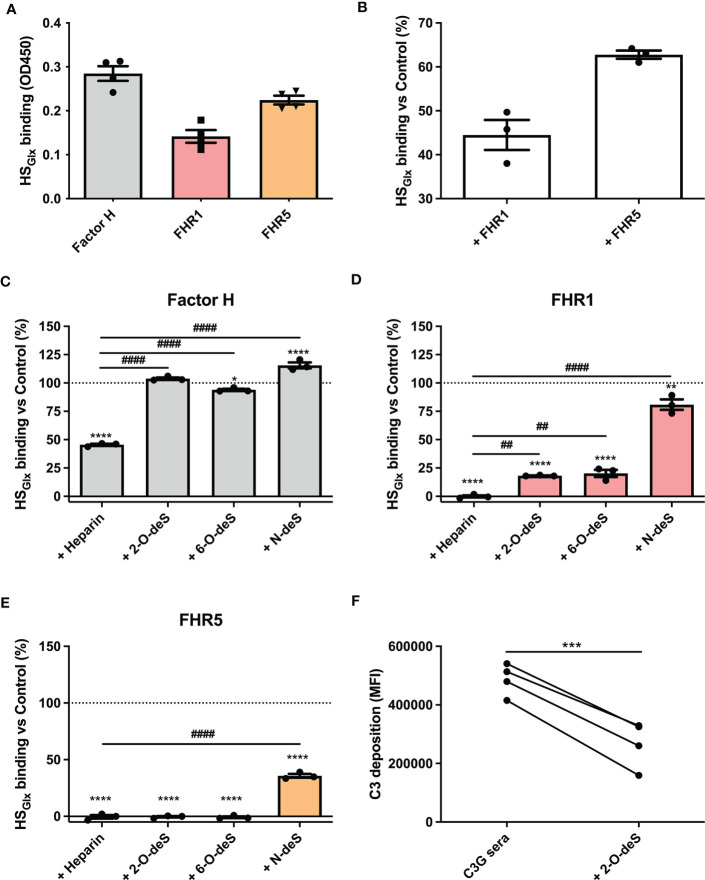
O-desulfated heparins reduce FHR1 and FHR5 binding to purified glomerular endothelial glycocalyx-derived heparan sulfate (HS), without affecting FH binding. HS was purified from isolated glycocalyx from conditionally immortalized glomerular endothelial cells (HS_Glx_) and immobilized on microtiter plates. Binding of factor H (FH) and factor H-related proteins (FHRs) was measured using ELISA (n=4) **(A)**. For competition assays, HS_Glx_ was incubated with FHR1 or FHR5 before binding of FH was determined (n=3) **(B)**. The contribution of specific sulfate modifications to HS_Glx_ ****binding of FH (n=3) **(C)**, FHR1 (n=3) **(D)** and FHR5 (n=3) **(E)** was evaluated by preincubating proteins with buffer (Control), heparin or 2-O-, 6-O- and N-desulfated (deS) heparin before addition to microtiter plates. To investigate potential therapeutic effects of 2-O-desulfated heparin in context of C3G patient sera (402H/H haplotype group with >1.5x increased C3 deposition compared to NHS), sera were supplemented with 50 µg/ml 2-O-desulfated heparin before addition to the cells (n=4) **(F)**. (*p < 0.05, **p < 0.01, ****p < 0.0001 *vs* Control; ^##^p < 0.01, ^####^p < 0.0001 *vs* Heparin; ***p<0.001 vs C3G sera.)

Since cells express other potential ligands for FH/FHR than only HS, we zoomed in on the interaction of FH and FHRs proteins with purified HS isolated from glycocalyx (HS_Glx_) of cultured glomerular endothelial cells. FH and FHRs binding to purified and immobilized HS_Glx_ revealed similar results as for binding to ciGEnCs, i.e. FH, FHR1 and FHR5 bound to HS_Glx_, whereas FHR2 did not (**Figure 3A** and **Supplementary Figure 2E**). Moreover, FHR1 and FHR5 efficiently competed with FH for binding to HS_Glx,_ since FH binding was reduced by the addition of FHR1 or FHR5 (**Figure 3B**).

### 2-O-Desulfated Heparin Oligosaccharides Selectively Inhibit FHR1 and FHR5 Binding, and Reduce C3b Deposition on Endothelial Cells

**Figure 4:**
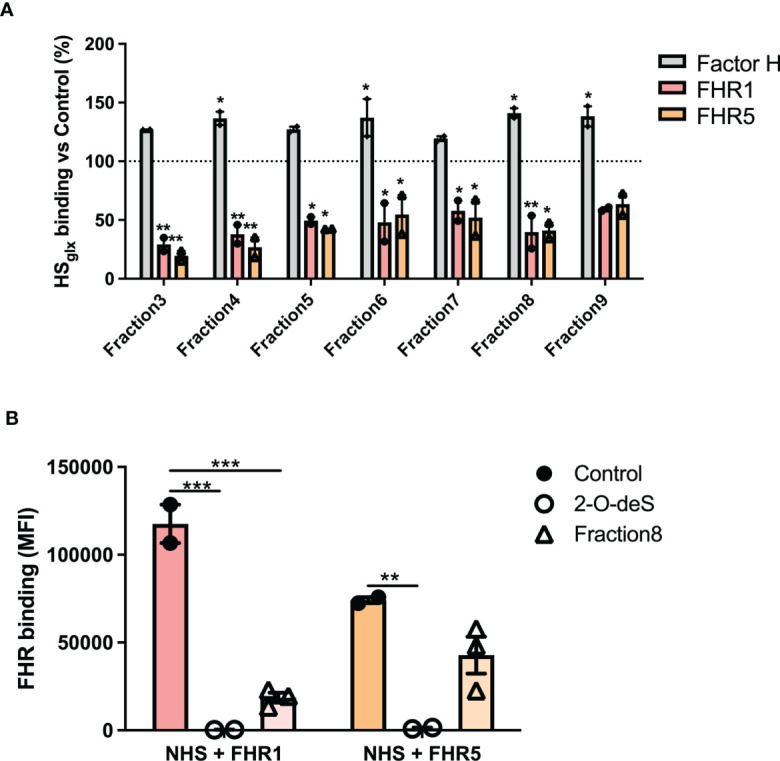
2-O-desulfated heparin fragments with a size equal to or larger than tetrasaccharides reduce FHR1 and 5 binding to purified glomerular endothelial HS_Glx_ and ciGEnCs, without affecting FH binding to HS_Glx_. To determine the minimal oligosaccharide size required for FHR1 and FHR5 competition, proteins were preincubated with equimolar amounts of 2-O-desulfated heparin digest size exclusion chromatography fractions (n=2) **(A)**. Addition of 2-O-deS heparin or short 2-O-deS oligosaccharides (Fraction8) to FHR-supplemented serum significantly reduced FHR binding to ciGEnCs (n≥2) **(B)**. (*p < 0.05, **p < 0.01, ***p < 0.001 *vs* Control).

To identify the nature of the HS structures in purified HS_Glx_ that are important for FH and/or FHR binding, we performed competition assays with heparin and N-, 2-O- or 6-O-de-sulfated heparins. Unmodified heparin blocked HS binding sites on FH, FHR1 and FHR5, thereby reducing (for FH) or preventing (for FHR1 and FHR5) binding to HS_Glx_, (**Figures 3C–E**). Removing any of the sulfates from heparin largely abolished the competition for FH binding to HS_Glx_ (**Figure 3C**). In contrast, 2-O- and 6-O-desulfated, and to a lesser degree, N-desulfated heparin, were still able to compete for FHR1 (**Figure 3D**) and FHR5 (**Figure 3E**). These results reveal that there is selectivity in binding of FH *versus* FHR1/FHR5 to structures within HS_Glx_. Importantly, C3b deposition by patient sera on ciGEnC was significantly decreased in the presence of 2-O-desulfated heparin, thereby suggesting that 2-O-desulfated heparin has therapeutic value for complement-mediated glomerular disease (**Figure 3F**). Addition of 2-O-desulfated heparin to FHR-supplemented serum reverse the C3b deposition caused by FHR1 competition with FH on ciGEnCs (**Supplementary Figure 4**) as well as on HUVECs (**Supplementary Figure 5**) in a concentration-dependent manner. Considering the highly heterogenous structure of HS and heparin, associated with many functions, a possible therapeutic should be preferably of short length whilst retaining the functional activity. Therefore, to determine the minimal size of 2-O-desulfated heparin required for restoring complement regulation, a size-defined library of oligosaccharides was generated from 2-O-desulfated heparin (**Supplementalry Figure 6** and **Supplementary Table 1**). Addition of size-defined 2-O-desulfated fractions revealed that a minimum of a tetrasaccharide (dp 4) was required for significant binding to FHR1 and FHR5 in competition with HS_Glx_ (**Figure 4A**), thereby reducing FHR binding to glomerular endothelial cells (**Figure 4B**). Importantly, FH binding to HS_Glx_ was not altered by any of the fractions tested (**Figure 4A**). Thus, 2-O-desulfated heparin oligosaccharides were identified as highly selective competitors reducing/preventing FHR1 and FHR5 binding to HS_Glx_, but not affecting FH binding to HS_Glx_, thereby supporting their potential application in novel C3G therapies.

## Discussion

The pathogenesis of C3G is driven by dysregulation of AP complement activity in the fluid phase and/or glomerular microenvironment. The relative balance between FH and type 1 FHR proteins is especially relevant in the glomerulus, as the latter compete with the former for host tissue-associated ligands, thereby affecting the relative degree of complement control in this microenvironment. One of the most important host ligands for FH and FHRs on glomerular endothelial cells is HS (36, 37).

In this study, we found that while FH, FHR1 and FHR5 bound to ciGEnCs, the initial deposition of C3b significantly increased this binding. The biggest relative changes were observed for FHR5, the binding of which significantly reduced binding of FH, creating a microenvironment in which AP activity was dysregulated, although terminal pathway activity was not observed. FHR1, in contrast, did not inhibit FH binding to ciGEnCs but nevertheless did lead to dysregulated AP activity and activation of the terminal pathway. FHR1-mediated dysregulation therefore may differ on cell surfaces that have been C3b-labeled as compared to cell surfaces on which C3 convertase has already formed. This difference suggests that FHR1 may support the formation of C3 convertase, as has been described for properdin (38, 39). Of note, we found that FHR2, which is also a type 1 FHR, hardly bound to ciGEnCs.

Consistent with the results we observed with FHR1 and FHR5, we also measured a significant increase in AP activity in the sera of C3G patients, including patients with 3 or 4 copies of the *CFHR1* gene. This increase in AP activity was not seen in C3G patients with Δ*CFHR3*-*CFHR1* deficiency. These findings suggest that in addition to fluid-phase dysregulation of the AP, which is characteristic of C3G, dysregulation of complement can also occur in the glomerular microenvironment and is impacted by the relative levels of FH and FHR1/FHR5 in the circulation.

To further clarify the importance of HS modifications, we studied the interaction between FH, FHRs and HS using purified HS_Glx_, *i.e.* in the absence of other cell surface ligands. In these experiments, we compared heparin and HS_Glx_ and found that the former competed more efficiently for FHR1 and FHR5 than for FH. Selectively desulfated heparin did not influence binding of FH to HS_Glx_, which shows that binding of FH to HS_Glx_ depends on N-, 2-O- and 6-O-sulfation. These results match previous reports involving FH-competition experiments with selectively desulfated heparins (9, 40). Selectively desulfated heparins were successful in preventing or reducing binding of FHR1 and FHR5 to HS_Glx_, except for N-desulfated heparin that still allowed FHR1 binding, which shows that FHR1 binding is primarily mediated by N-sulfation.

Notably, although FHR5 bound more strongly to HS_Glx_ as compared with FHR1, FHR1 was the more potent competitor for FH binding to HS_Glx_. This difference between FHR1 and FHR5 reflects the close homology between the HS binding domains of FHR1 (CCP5) and FH (CCP20), which share 97% identity. The two variant amino acids–L290 *vs* S1191 and A296 *vs* V1197–in FH and FHR1 are buried and therefore unlikely to interact directly with HS. They do however affect the structure of the surface-exposed loop that harbors the HS-binding amino acids K285/K1186 and K287/K1188 in FHR1 and FH, respectively (41), possibly altering surface charge distribution and consequently the HS binding characteristics of the domain. This remarkable degree of ligand specificity is also seen with age-related macular degeneration and the associated p.Tyr402His polymorphism (rs1061170) in CCP7 of FH, which affects FH binding to specific HS modifications in Buch’s membrane and is a risk factor for disease (26). Type 1 FHRs (FHR1, 2 and 5) can also exist as heterodimers or homodimers, which we have not addressed in this study. However, we do not consider this as a major limitation, since homodimers of FHR1, 2 and 5 may have formed in our assays. Most likely, our primary finding that 2-O-desulfated heparin can prevent binding of FHR1 and FHR5 is also valid for heterodimers, since the monomers present in a heterodimer both will bind to 2-O-desulfated heparin, whereas FHR2 hardly binds to HS_Glx_.

The ability of 2-O-desulfated heparin oligosaccharides to compete for FHR but not FH and thereby alter binding to HS_Glx_ suggests that providing a “sink” to scavenge FHR proteins represents a novel treatment approach for C3G. 2-O-desulfation by alkaline lyophilization is not only remarkably selective and simple, but it also removes the rare glucosamine 3-O-sulfate modification, which significantly reduces the anticoagulant activity of 2-O-desulfated heparin (42) and thus potential adverse effects. We observed that selective competition is retained with short 2-O-desulfated oligosaccharides (≥tetrasaccharides), potentially enabling the synthesis of therapeutically active oligosaccharides by chemoenzymatic methods (43).

**Figure 5:**
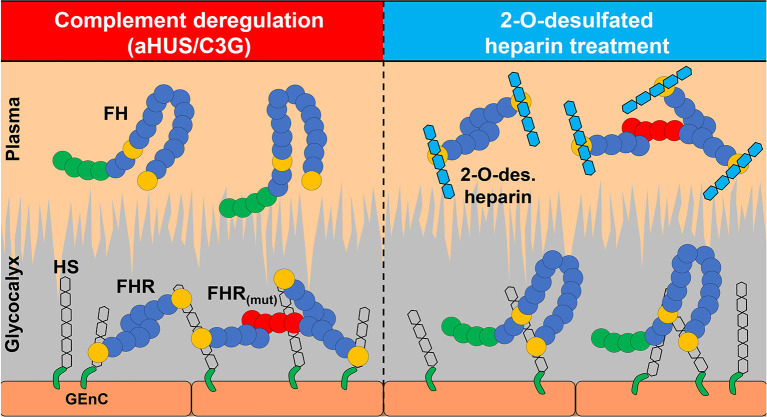
2-O-desulfated heparin oligosaccharides are potential therapeutics for C3G and other diseases with dysregulated complement. Pathogenic changes in complement components or glomerular glycocalyx affect the balance between FH and FHRs contributing to disease development. 2-O-desulfated heparin derivatives selectively inhibit FHR1 and FHR5 binding to glomerular endothelium without affecting FH binding, which makes them potential therapeutics for C3G and other diseases with dysregulated complement.

In conclusion, we have demonstrated HS-mediated ligand specificity for FH and FHR1/FHR5 that impacts the relative balance of these proteins in the glomerular glycocalyx, thereby altering AP regulation in this microenvironment. These results provide novel insights into the pathophysiology of C3G and suggest that genetic studies of C3G cohorts can identify patients with variants in genes involved in HS proteoglycan synthesis that create a “permissive” microenvironment, which favors FHR binding over FH binding. This imbalance, in turn, supports complement dysregulation either primarily or after secondary triggering events. Our data also suggest a novel treatment for C3G in which short 2-O-desulfated heparin oligosaccharides are used to scavenge FHR1 and FHR5, thereby altering the binding of these proteins to the glomerular glycocalyx (**Figure 5**).

## Data Availability Statement

The raw data supporting the conclusions of this article will be made available by the authors, without undue reservation.

## Ethics Statement

The study was approved by the Institutional Review Board of Carver College of Medicine at the University of Iowa. Written informed consent to participate in this study was provided by the participants’ legal guardian/next of kin.

## Author Contributions

JvdV initiated and supervised the study. ML and JvdV designed the study. MMH, ML, CY, MCH, MdG and EK carried out experiments. MMH, ML, CY and MCH analyzed the data and made the figures. MMH, ML, CY, MCH, EK, TvK, JW, TJR, RS and JvdV drafted and revised the manuscript. All authors contributed to the article and approved the submitted version.

## Funding

This study was supported by the Radboud university medical center PhD fellow program, consortium grant LSHM16058-SGF (GLYCOTREAT, a collaboration project financed by the PPP Allowance made available by Top Sector Life Sciences & Health to the Dutch Kidney Foundation to stimulate public-private partnerships), Kidneeds, GCR foundation USA JV and the National Institutes of Health R01 DK110023 RS.

## Conflict of Interest

Author EK was employed by company Aspen API.

The remaining authors declare that the research was conducted in the absence of any commercial or financial relationships that could be construed as a potential conflict of interest.

## Publisher’s Note

All claims expressed in this article are solely those of the authors and do not necessarily represent those of their affiliated organizations, or those of the publisher, the editors and the reviewers. Any product that may be evaluated in this article, or claim that may be made by its manufacturer, is not guaranteed or endorsed by the publisher.
