# Supplementary material for: Selective Binding of Heparin/Heparan Sulfate Oligosaccharides to Factor H and Factor H-Related Proteins: Therapeutic Potential for C3 Glomerulopathies
Source: Front Immunol. 2021 Aug 18;12:676662. doi: 10.3389/fimmu.2021.676662 (PMC8416517; doi:10.3389/fimmu.2021.676662)
Supplement: Supplementary file 1 [file DataSheet_1.docx]

Supplementary Material

**Supplementary Figure 1. The classical pathway does not contribute to C3b deposition in the alternative pathway activity assays.** Conditionally immortalized glomerular endothelial cells were either left untreated (Untr.) or sensitized with anti-Jurkat/Ramos/THP-1 antiserum (AB) to induce classical pathway activation. Cells were incubated with 20% human serum in veronal-buffered saline supplemented with EGTA or Hank's balanced salt solution including Mg2+/Ca2+ (HBSS), i.e. buffers which prevent and enable classical pathway activation, respectively. C3b deposition was compared to determine if the classical pathway contributes significantly to the C3b deposition observed in the AP activity assays (n=2) (****p<0.0001 vs Untr./EGTA; MFI: Mean fluorescence intensity).

**Supplementary Figure 2. FHR2 shows minimal binding to ciGEnCs, but increases C3b deposition on ciGEnCs.** C3b deposition induced by the classical pathway increased FHR2 binding to ciGEnCs (n=3) (A). The alternative pathway activity as measured by C3b deposition on ciGEnCs was further increased by the presence of FHR2 (n=3) (B), but FHR2 did not affect the terminal pathway (n=3) (C). FHR2 did not affect FH binding to ciGEnCs (n=4) (D), and did not display binding to HS_Glx_ (n=4) (E).

**Supplementary Figure 3. Measurement of C3b deposition analyzed via flow cytometry and Western blot align.** Cell lysates of ciGEnCs with NHS, or with NHS with EDTA, which inhibits C3b deposition, were resolved by SDS PAA gel electrophoresis, blotted and probed with anti-C3b antibodies (A). Cells with NHS, or with NHS with EDTA, which inhibits C3 deposition, were detached and analyzed by flow cytometry (B). Quantitative analysis of both Western blot assay and flow cytometry assay revealed a comparable result (C).

**Supplementary Figure 4. 2-O-desufated heparin induced inhibition of C3b deposition on ciGEnCs is concentration dependent for FHR1 and FHR5.** Increasing concentrations of 2-O-desulfated heparin (2-O-deS) were incubated with (FHR-supplemented) serum before addition to the ciGEnCs (n=2).

**Supplementary Figure 5. 2-O-desulfated heparin reverses FHR1- and FHR5-mediated alternative pathway deregulation on human umbilical vein endothelial cells.**

Human umbilical vein endothelial cells were incubated in 20% normal human serum (NHS) in veronal-buffered saline including 5 mM magnesium-EGTA, which prevents classical/lectin pathway activation. Serum was supplemented with factor H-related proteins (FHRs) 1 or 5, or FHR proteins including 2-O-desulfated (2-O-des.) heparin before addition to the cells. Effects on alternative pathway activity were evaluated using flow cytometry (n=2) (***p<0.001, ****p<0.0001 vs Untreated).

**Supplementary Figure 6.** **2-O-desulfated heparin oligosaccharide library generation.** 2-O-desulfated heparin was digested into oligosaccharides by heparinase II (A) and the oligosaccharides were separated into different size fractions (Fraction1-9) using size exclusion chromatography (SEC) (B), representative SEC chromatogram of digested 2-O-desulfated heparin. AU, absorbance units. Size range and resolution of SEC fractions were resolved by gel electrophoresis and visualized by silver staining compared to (Arixtra®, dp5), and heparin size defined oligosaccharides: decasaccharides (dp10) and docosasaccharide (dp22) (C).

**Supplementary Table 1.** **Alkaline lyophilization selectively reduces 2-O-sulfation of heparin*.**

|  | **Heparin (%)** | **2-O-desulf.**  **heparin (%)** |
| --- | --- | --- |
| ΔUA,2S-GlcNS,6S | 69.65 | 2.87 |
| ΔUA,2S-GlcNAc,6S | 1.69 | 0.24 |
| ΔUA-GlcNS,6S | 11.01 | 76.09 |
| ΔUA-GlcNAc,6S | 2.93 | 4.49 |
| ΔUA,2S-GlcNS | 7.47 | 0.67 |
| ΔUA,2S-GlcNAc | 1.79 | 0.15 |
| ΔUA-GlcNS | 2.88 | 11.31 |
| ΔUA-GlcNAc | 2.58 | 4.19 |

*Disaccharide profiles of 2-O-desulfated (2-O-desulf.) heparin and the original heparin preparation. The relative amount of 2-O-sulfated disaccharides in 2-O-desulfated heparin was selectively reduced from 81% to 4% compared to the original material, whereas N- and 6-O-sulfates were essentially unaffected (91% to 91% and 85% to 84%, respectively (Orange: 2-O-sulfated disaccharides, Green: Disaccharides lacking 2-O-sulfates; ΔUA: unsaturated uronic acid, GlcNAc: N-acetylglucosamine, GlcNS: N-sulfoglucosamine, 2S: 2-O-sulfation, 6S: 6-O-sulfation).
