# Supplementary figures and images for: Selective Binding of Heparin/Heparan Sulfate Oligosaccharides to Factor H and Factor H-Related Proteins: Therapeutic Potential for C3 Glomerulopathies
Source: Front Immunol. 2021 Aug 18;12:676662. doi: 10.3389/fimmu.2021.676662 (PMC8416517; doi:10.3389/fimmu.2021.676662)

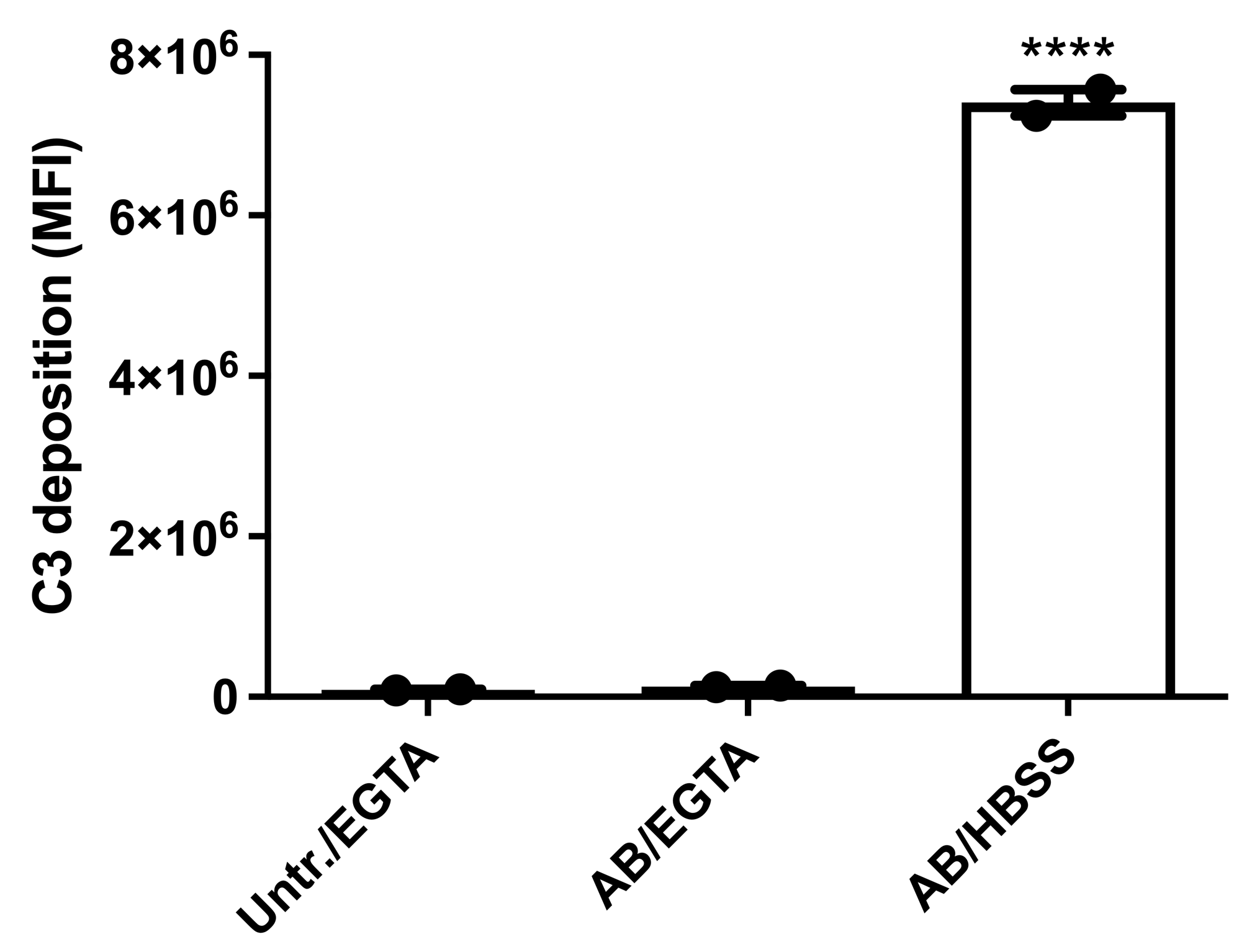

Supplement: Supplementary file 2 [file Image_1.tiff]

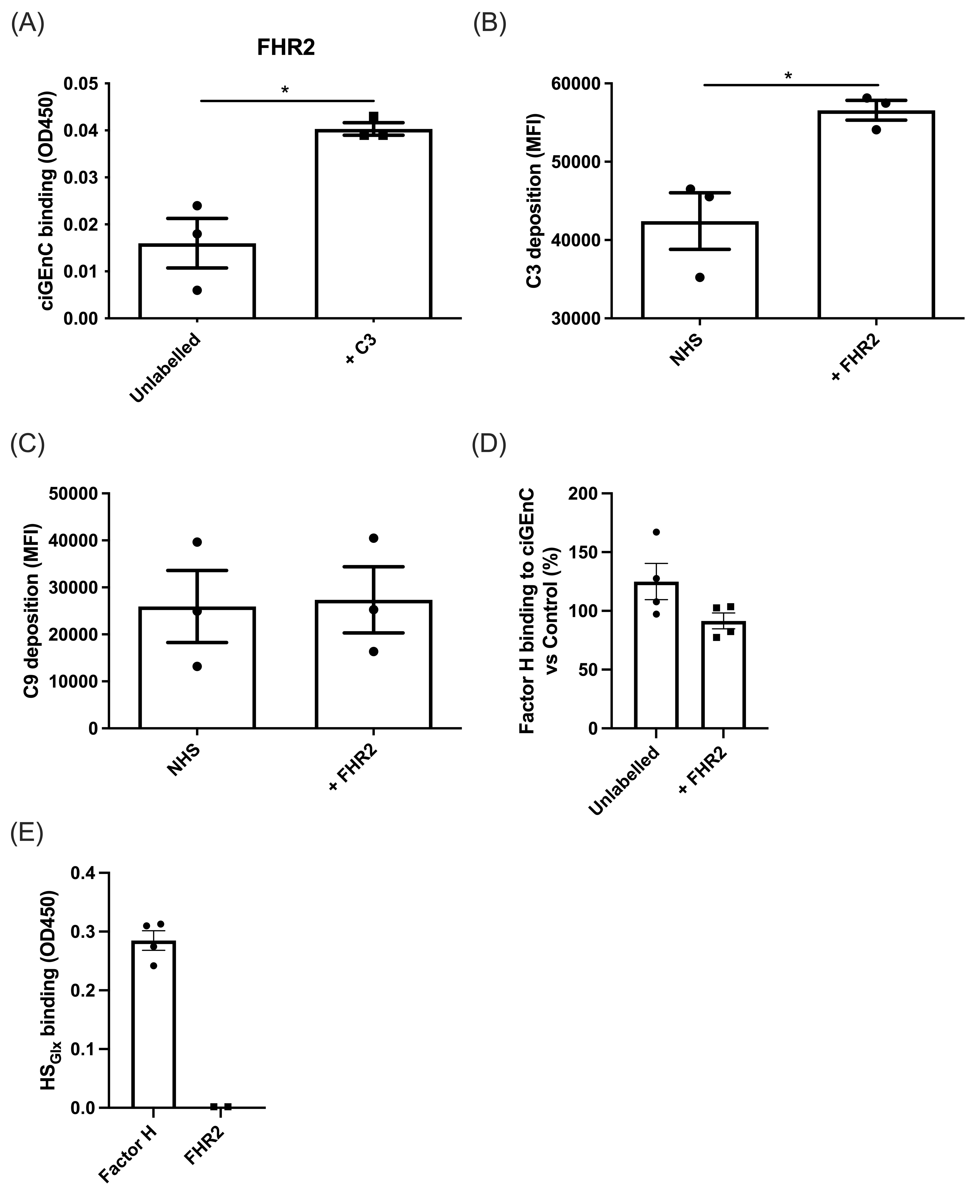

Supplement: Supplementary file 3 [file Image_2.tiff]

(A)

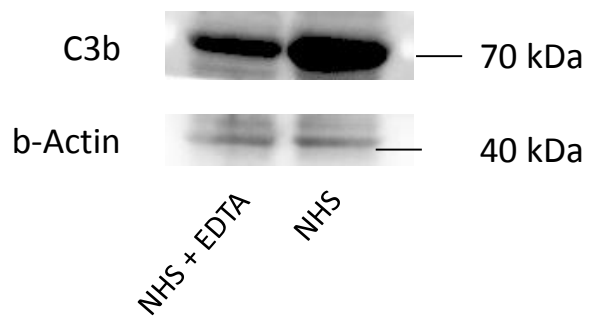

(B)

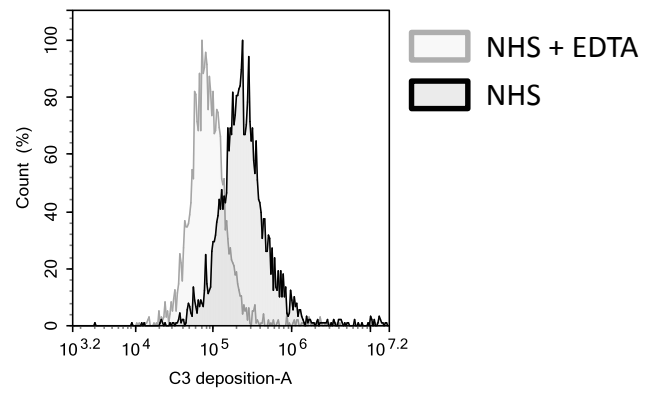

(C)

| Sample     | Area - WB | MFI - FCM |
|------------|-----------|-----------|
| NHS + EDTA | 9734.681  | 37.346    |
| NHS        | 16601.045 | 83.064    |
| Ratio      | 1.7       | 2.2       |

Supplement: Supplementary file 4 [file Image_3.pdf]

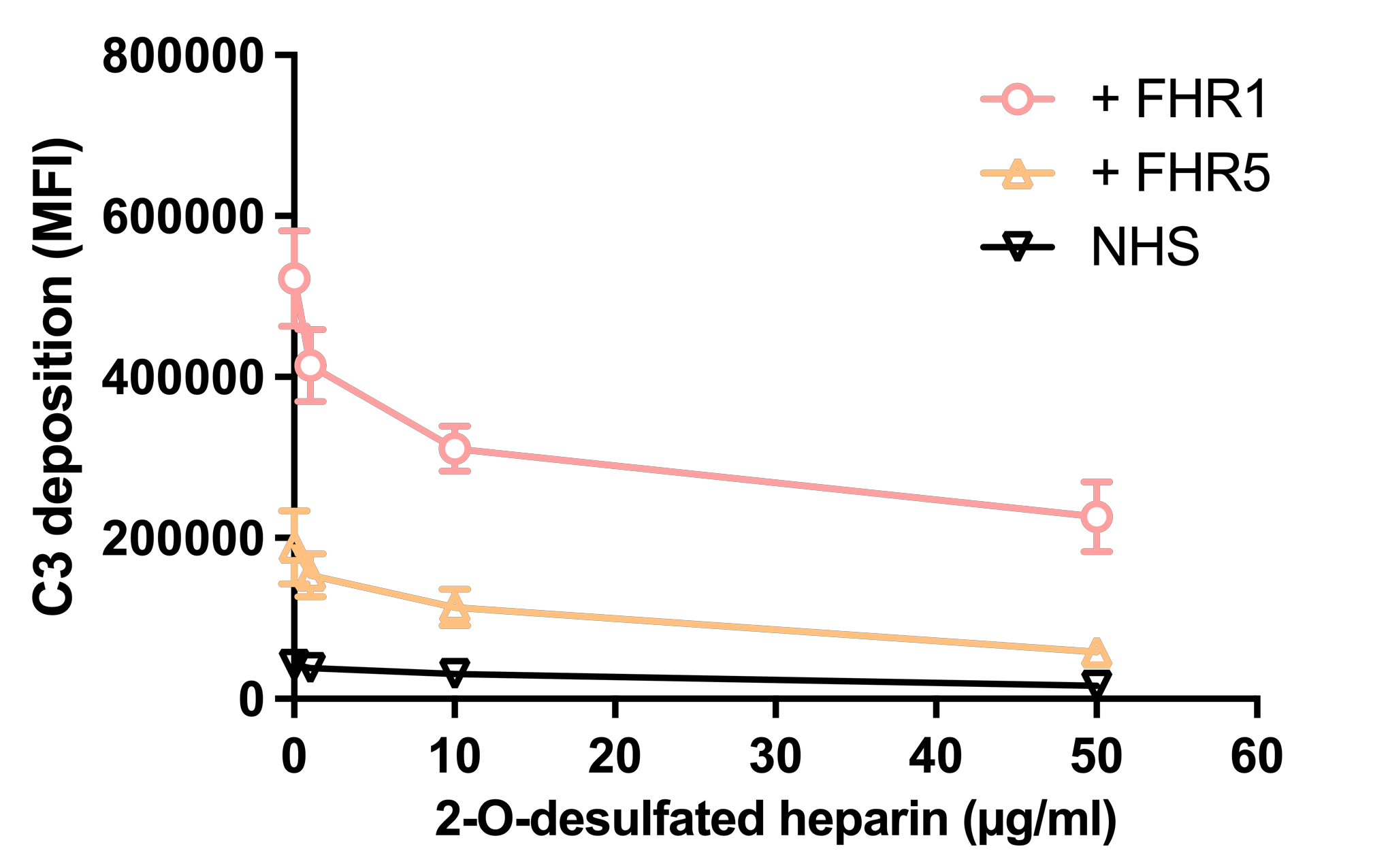

Supplement: Supplementary file 5 [file Image_4.tiff]

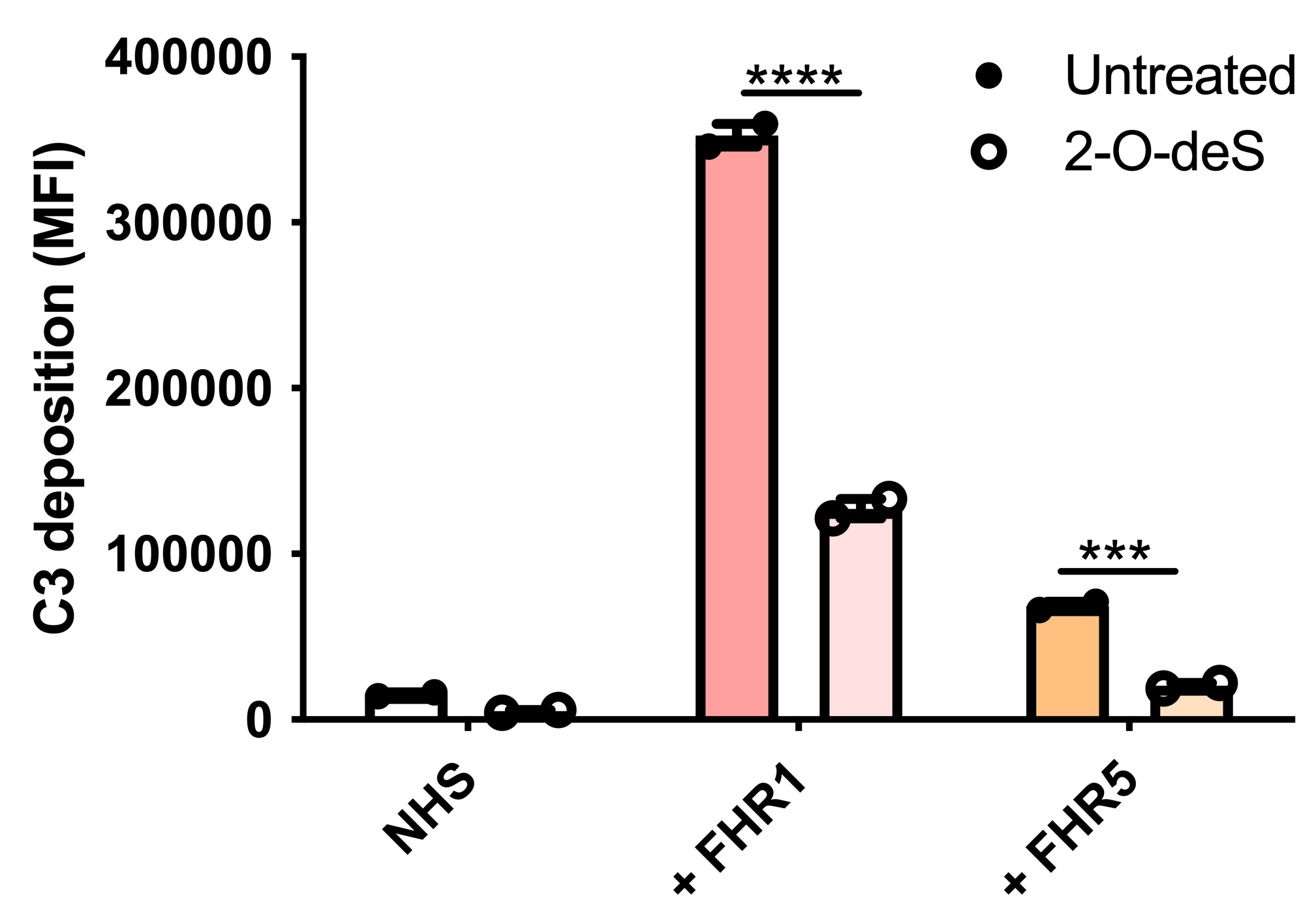

Supplement: Supplementary file 6 [file Image_5.tiff]

(A)

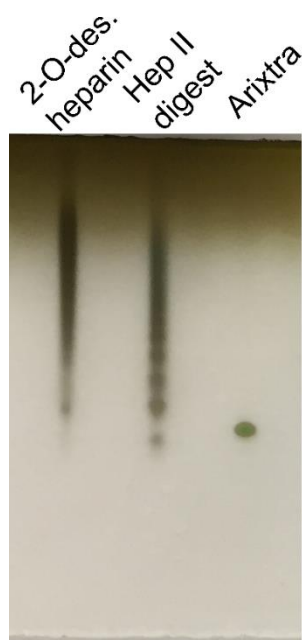

(C)

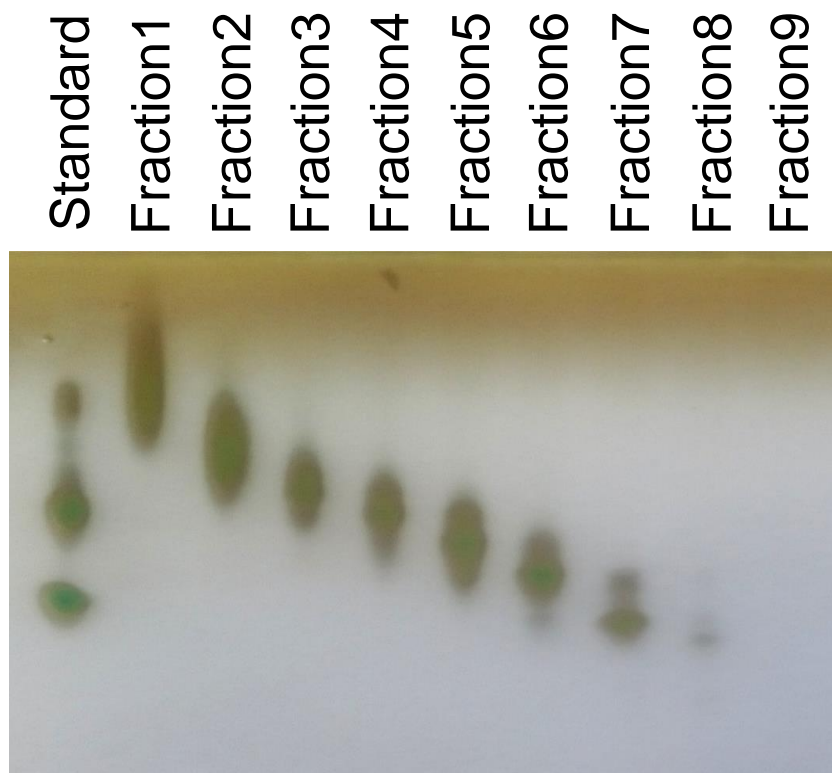

(B)

Fraction1  
Fraction2  
Fraction3  
Fraction4  
Fraction5  
Fraction6  
Fraction7  
Fraction8  
Fraction9

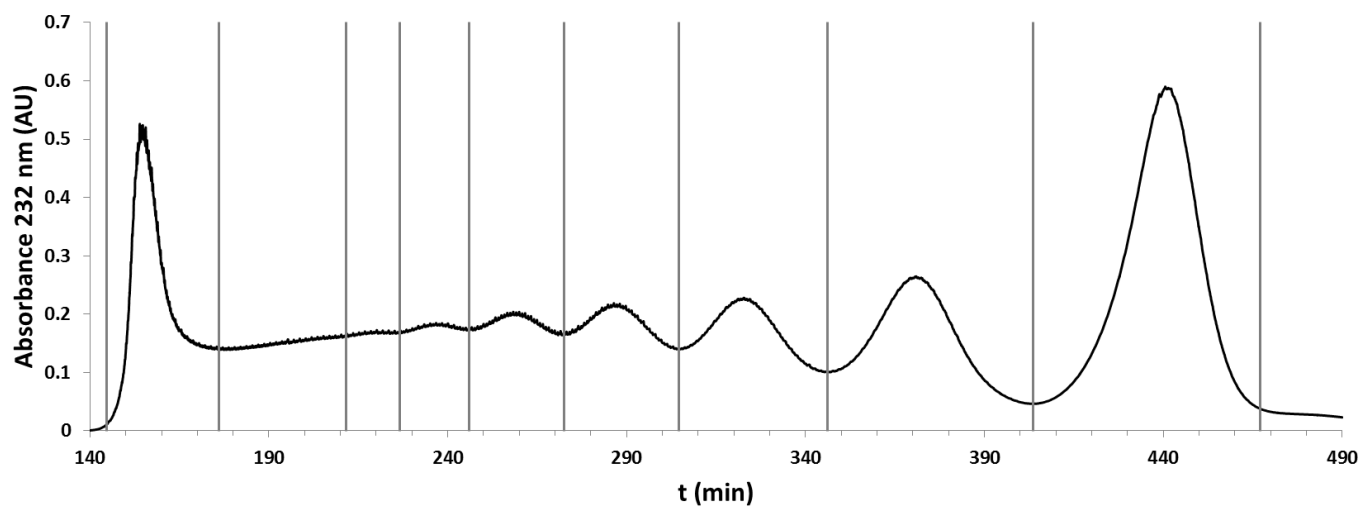

Supplement: Supplementary file 7 [file Image_6.pdf]
